# Fine-Scale Structure Analysis Shows Epidemic Patterns of Clonal Complex 95, a Cosmopolitan *Escherichia coli* Lineage Responsible for Extraintestinal Infection

**DOI:** 10.1128/mSphere.00168-17

**Published:** 2017-05-31

**Authors:** David M. Gordon, Sarah Geyik, Olivier Clermont, Claire L. O’Brien, Shiwei Huang, Charmalie Abayasekara, Ashwin Rajesh, Karina Kennedy, Peter Collignon, Paul Pavli, Christophe Rodriguez, Brian D. Johnston, James R. Johnson, Jean-Winoc Decousser, Erick Denamur

**Affiliations:** a Ecology and Evolution, Research School of Biology, the Australian National University, Acton, Australian Capital Territory, Australia; b UMR 1137 INSERM and Université Paris Diderot, IAME, Sorbonne Paris Cité, Paris, France; c Medical School, Australian National University, Canberra, Australian Capital Territory, Australia; d Gastroenterology and Hepatology Unit, Canberra Hospital, Canberra, Australian Capital Territory, Australia; e Department of Botany, Faculty of Science, University of Peradeniya, Peradeniya, Sri Lanka; f Infectious Disease and Microbiology, Canberra Hospital, Woden, Australian Capital Territory, Australia; g ACT Pathology, Canberra, Australian Capital Territory, Australia; h UMR 955 INSERM and Université Paris-Est, Assistance Publique-Hôpitaux de Paris, Hôpital Henri Mondor, Créteil, France; i VA Medical Center and Department of Medicine, University of Minnesota, Infectious Diseases, Minneapolis, Minnesota, USA; j UMR 1137, INSERM, IAME, Paris and Assistance Publique-Hôpitaux de Paris, Hôpital Henri Mondor, Créteil, France; k Assistance Publique: Hôpitaux de Paris, Hôpital Bichat, Paris, France; Centers for Disease Control and Prevention

**Keywords:** clonal complex 95, *Escherichia coli*, epidemiology, population genetics

## Abstract

The *Escherichia coli* lineage known as clonal complex 95 (CC95) is a cosmopolitan human-associated lineage responsible for a significant fraction of extraintestinal infections of humans. Whole-genome sequence data of 200 CC95 strains from various origins enabled determination of the CC95 pangenome. The pangenome analysis revealed that strains of the complex could be assigned to one of five subgroups that vary in their serotype, extraintestinal virulence, virulence gene content, and antibiotic resistance gene profile. A total of 511 CC95 strains isolated from humans living in France, Australia, and the United States were screened for their subgroup membership using a PCR-based method. The CC95 subgroups are nonrandomly distributed with respect to their geographic origin. The relative frequency of the subgroups was shown to change through time, although the nature of the changes varies with continent. Strains of the subgroups are also nonrandomly distributed with respect to source of isolation (blood, urine, or feces) and host sex. Collectively, the evidence indicates that although strains belonging to CC95 may be cosmopolitan, human movement patterns have been insufficient to homogenize the distribution of the CC95 subgroups. Rather, the manner in which CC95 strains evolve appears to vary both spatially and temporally. Although CC95 strains appeared globally as pandemic, fine-scale structure analysis shows epidemic patterns of the CC95 subgroups. Furthermore, the observation that the relative frequency of CC95 subgroups at a single locality has changed over time indicates that the relative fitness of the subgroups has changed.

**IMPORTANCE** *Escherichia coli* clonal complex 95 represents a cosmopolitan, genetically diverse lineage, and the extensive substructure observed in this lineage is epidemiologically and clinically relevant. The frequency with which CC95 strains are responsible for extraintestinal infection appears to have been stable over the past 15 years. However, the different subgroups identified within this lineage have an epidemic structure depending on the host, sample, continent, and time. Thus, the evolution and spread of strains belonging to CC95 are very different from those of another cosmopolitan human-associated clonal complex, CC131, which has increased significantly in frequency as a cause of extraintestinal infection over the past 15 years due to the evolution and spread of two very closely related, nearly monomorphic lineages.

## Abstract (precis)

*Escherichia coli* clonal complex 95 represents a cosmopolitan, genetically diverse lineage, and the extensive substructure observed in this lineage is epidemiologically and clinically relevant. The frequency with which CC95 strains are responsible for extraintestinal infection appears to have been stable over the past 15 years. However, the different subgroups identified within this lineage have an epidemic structure depending on the host, sample, continent, and time. Thus, the evolution and spread of strains belonging to CC95 are very different from those of another cosmopolitan human-associated clonal complex, CC131, which has increased significantly in frequency as a cause of extraintestinal infection over the past 15 years due to the evolution and spread of two very closely related, nearly monomorphic lineages.

## INTRODUCTION

Many of the bacteria that cause disease in humans represent geographically widespread, human-specific, nearly monomorphic lineages within species that are much more genetically diverse (1). High-throughput sequencing technologies now allow investigation of the population structure and evolution of these pathogens (2–6). Such studies have revealed that these pathogens are relatively new, with their most recent common ancestor existing from 3,000 to 500 years ago. These studies have also revealed where some of these pathogens originated, how they have spread geographically, and, in some instances, how they have acquired resistance to antimicrobial agents.

*Escherichia coli* is a genetically diverse species that is primarily a commensal of the lower intestinal tract of mammals and, to a lesser extent, birds. However, particular lineages of *E. coli* are well-known intestinal pathogens that cause diarrhea, most notably the enterohemorrhagic strain O157:H7. Additionally, *E. coli* is the most common cause of urinary tract infection and a leading cause of bloodstream infection. Although *E. coli* isolates from extraintestinal sites in humans include diverse strains, a very significant fraction represent just a few lineages (7, 8). Indeed, the great majority belong to fewer than two dozen clonal complexes (CCs), namely, those represented by sequence type 10 (ST10 [phylogroup A]), STs 12, 14, 73, 95, 127, 131, 141, 144, and 372 (all phylogroup B2), and ST69 (phylogroup D). However, most extraintestinal isolates belong to just one of four well-defined CCs, CC73, -95, -131, and -69, which are geographically widespread, if not cosmopolitan (8). There is a well-developed understanding of the epidemiology and genetic structure of CC131 (9, 10), as members of the complex have, in recent years, become one of the most frequent causes of extraintestinal infection by *E. coli*. In contrast, little is known about members of CC95, -73, or -69.

The present study focuses on strains belonging to CC95, a cosmopolitan lineage that in previous studies has been responsible for, on average, 17% of extraintestinal infections in humans caused by *E. coli* (11–17). We used whole-genome sequence (WGS) data and PCR analysis to investigate a large collection of CC95 isolates from diverse time periods, locales, hosts, and contexts (i.e., commensal versus infection isolates). The results of this analysis reveal that the diversity to be found within this clonal complex varies geographically, temporally, and with the strain’s source of isolation (e.g., fecal versus extraintestinal) and the sex of the host (for human isolates).

## RESULTS

### Distribution and abundance of CC95: host range of CC95.

The continent of Australia is home to diverse vertebrate species. The human population is sparsely distributed (3.06 people km^−2^) and highly urbanized (89%). Europeans occupied Australia beginning in 1788, and since European settlement, levels of sanitation have been relatively high. Consequently, Australia represents an ideal location in which to determine the diversity of vertebrate species in which CC95 may be found.

Large-scale surveys of *E. coli* from a variety of Australian vertebrate hosts have shown that *E. coli* can be recovered from any vertebrate host group (18, 19). However, strains belonging to CC95 have not been detected in the feces of any sampled native mammal (*n =* 1,063), backyard poultry (*n =* 278), amphibian (*n =* 106), or fish (*n =* 138); likewise, a CC95 strain was recovered from only 1 of 447 reptiles and 1 of 1,312 native birds. Overall, the probability of detecting a CC95 strain in the feces of a nonhuman “native” vertebrate is less than 0.06%. CC95 strains were recovered from 5.6% of 306 Australian samples of commercial poultry meat (D. M. Gordon and B. Vangchhia, unpublished data).

**FIG 1:**
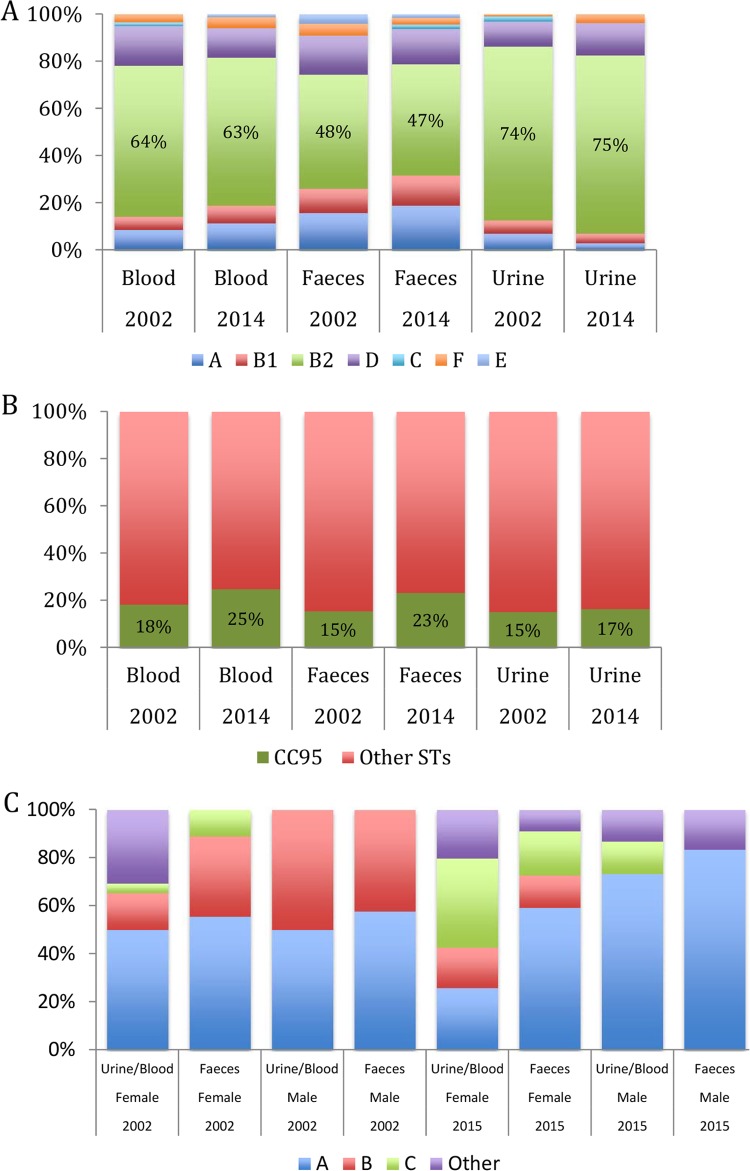
Relative frequency of phylogroups, CC95 isolates, and CC95 subgroups among *E. coli* strains recovered from feces, urine, or blood samples taken from people living in Canberra in 2002 and 2014. (A) Relative frequency of phylogroups among *E. coli* isolates; (B) relative frequency of CC95 strains among B2 strains; (C) Relative frequency of CC95 subgroups among CC95 strains.

Although members of CC95 are rare in nonhuman vertebrates, in humans, CC95 isolates are not only frequently observed, but their frequency appears to be temporally stable. Two large collections of *E. coli* strains collected from human feces, urine, and blood by the Microbiology Laboratory of the Canberra Hospital, Australia, in 2002 (20) and again in 2014 and 2015 using nearly identical methods were assigned to a phylogroup and screened for members of CC95 as described in Materials and Methods. Although the frequency of phylogroup B2 strains was highest among urine isolates and lowest among fecal isolates, with blood isolates intermediate, there was no change in the relative abundance of phylogroup B2 strains between 2002 and 2014, irrespective of the isolate’s source (feces, urine, or blood) (nominal logistic regression: year, *P* = 0.98; source, *P* < 0.001; year-source interaction, *P* = 0.86) (Fig. 1A). Among the B2 strains, the CC95 strains represented 19% of the isolates, but here again, there was no significant change in the frequency of CC95 isolates between 2002 and 2014 and no difference in the frequency of CC95 isolates with respect to the isolate’s source (nominal logistic regression: year, *P* = 0.07; source, *P* = 0.26; year-source interaction, *P* = 0.67) (Fig. 1B).

### CC95 pangenome analysis. (i) The pangenome.

**FIG 2:**
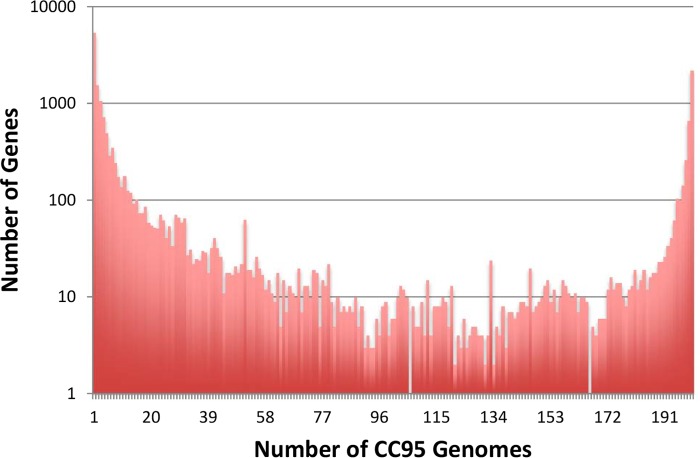
Distribution of the genes in the pangenome of *E. coli* CC95. The number of genes found in 1 to 200 strains, respectively, is shown. All genomes were annotated using Prokka, and the pangenome was determined using ROARY.

There were a total of 17,603 genes among 200 CC95 strains recovered from feces and extraintestinal sites at a variety of geographic locations. The core genome (present in ≥99% of strains) consisted of 3,134 genes: 534 genes were found in from 95% to <99% of strains, 1,196 were found in 30% to <95% of strains, and 11,934 were found in fewer than 15% of the strains. Over 5,400 genes were found in only a single isolate, and 2,210 genes were found in all 200 isolates, with the balance of the genes being present in >1 or <200 isolates, giving rise to a U-shaped distribution for the number of isolates in which a gene is present (Fig. 2).

### (ii) Core genome variation.

**FIG 3:**
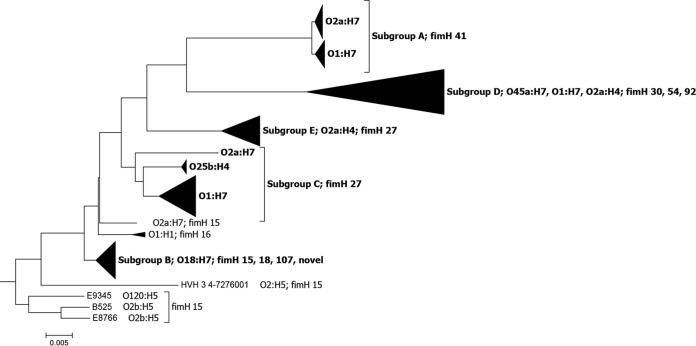
Substructure of 200 *E. coli* CC95 strains. Group B2 strain ED1a was used as an outgroup (not shown). The single nucleotide polymorphisms (SNPs) were detected using the Harvest Suite (43) of tools and ED1a as the reference strain. Gubbins (53) was used to infer recombination events, and recombinant sites were removed. A maximum likelihood tree was inferred with a general time-reversible (GTR) model of evolution using MEGA 6.0 (54).

The phylogeny inferred for these 200 CC95 isolates revealed, as expected, significant structure within CC95, with the observed clusters correlating largely with a strain’s serotype (Fig. 3). Five main clusters were evident. The cluster of strains with an O1:H7 or an O2a:H7 serotype was designated subgroup A. The cluster of strains with an O18:H7 serotype, including UTI89, was designated subgroup B. The cluster of O1:H7 strains, which also included O25b:H4 and O2a:H7 strains, was designated subgroup C. The cluster of strains that included those with O45a:H7, O1:H7, and O2a:H4 serotypes was designated subgroup D. The cluster of strains with an O2a:H4 serotype was designated subgroup E. Additionally, 9 (5%) of the 200 strains examined did not fall into one of these five subgroups, and 4 of these strains with the H5 antigen and *fimH* allele 15 were distinct from the other CC95 strains (Fig. 3). Within four of the five CC95 subgroups, strains that shared the same serotype also tended to share the same *fimH* allele (21). The sole exception was the subgroup B strains, which despite all being O18:H7 exhibit four different *fimH* alleles (Fig. 3).

Subgroup D comprised strains of diverse serotypes. However, there seemed to be little value in further subdividing subgroup D, as all of the subgroup D strains were separated from the other subgroups by long branches. Additionally, a practical reason for not subdividing subgroup D is that although CC95 strains are relatively common, they will never represent a large fraction of isolates in any strain collection, and smaller sample sizes mean less statistical power.

### (iii) Variable gene content.

**FIG 4:**
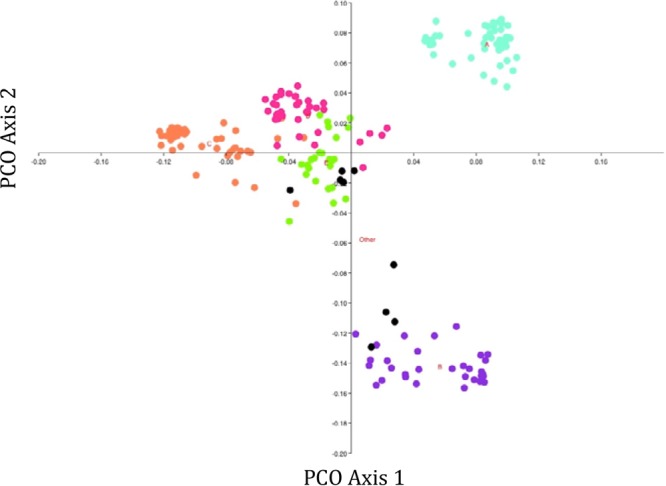
Similarity among *E. coli* CC95 isolates based on the variable genome. Genes present in all genomes or in a single strain were eliminated. Among-strain similarity was quantified using a Jaccard metric, and the dimensions of the matrix were reduced using principal-coordinate (PCO) analysis as implemented in PAST. Turquoise denotes subgroup A strains, purple B strains, orange C strains, pink D strains, green E strains, and black unassigned strains.

Analysis of variable genes showed that the substructure revealed by the core genome phylogeny was reflected in the variable gene content of the strains (Fig. 4). Subgroups A, B, and C had variable gene contents that were distinct from those of the other phylogroups. The subgroup E strains were an exception, since despite being tightly clustered and distinct according to the core genome, they showed some variable gene content overlap with subgroup D strains.

Multiple genes within the variable genome have been identified as potentially enhancing a strain’s propensity to cause extraintestinal disease or colonize a host (22, 23). *In silico* analysis of 40 such virulence factors among the 200 CC95 isolates showed that *afdA*, as well as *focG*, *lpfA*, and *terC*, is absent from all strains (see Table S1 in the supplemental material). In contrast, all CC95 strains have *fimH*, *fyuA*, and *ompT*. Between these extremes, less than 5% of CC95 strains have the gene *sfa*, *iha*, or *tsh* or harbor the plasmid encoding microcin B17 or colicins B and M, whereas more than 95% have the virulence genes *neuC*, *sitA*, *usp*, and *vat*. Other virulence genes have midrange prevalence values.

10.1128/mSphere.00168-17.1 TABLE S1  CC95 subgroup assignment, source, country, and year of isolation, together with the strain’s virulence factor and antimicrobial and resistance gene profile. Download TABLE S1, XLS file, 0.1 MB. Copyright © 2017 Gordon et al. 2017 Gordon et al. This content is distributed under the terms of the Creative Commons Attribution 4.0 International license.

As with serotype, the presence of many virulence genes varied in relation to the subgroup membership of the CC95 strain (Table 1). Overall, subgroup A strains possessed the colibactin gene cluster, the self-adhesion locus antigen 43, and the toxins *tcpC* and *senB*. Subgroup B strains also have the colibactin locus, but in addition possessed the invasion of brain epithelium gene *ibeA* and were likely to have the invasion determinant *tia* and the toxin determinants *cnf1*, *hylD*, and *cdiA*. Relative to strains belonging to subgroups A and B, strains belonging to subgroups C, D, and E had more similar extraintestinal virulence factor profiles, but differed from subgroup A and B strains in having the plasmid-encoded bacteriocins colicin Ia and microcin V. Subgroup C, D, and E strains were also very likely to possess the iron-uptake-related genes, *ireA*, *iroN*, and *iucAC*. Subgroup C strains typically hosted a colicin E1 plasmid, while subgroup D and E strains did not. Most subgroup D strains possessed antigen 43, while subgroup C and E strains did not.

**TABLE 1:** Distribution of putative virulence factors among strains belonging to the five CC95 subgroups

Gene	% in CC95 subgroup:					*P* value^a^
	A (*n =* 51)	B (*n =* 31)	C (*n =* 48)	D (*n =* 38)	E (*n =* 25)	
*clbB*	100	100	4	0	44	<0.001
*cnf1*	0	58	0	0	0	<0.001
Colicin E1 gene	0	0	81	8	44	<0.001
Colicin Ia gene	24	23	83	68	52	<0.001
*etsC*	24	39	87	74	36	<0.001
*hylD*	0	58	0	13	0	<0.001
*ibeA*	0	100	2	0	0	<0.001
*ireA*	90	13	100	95	100	<0.001
*iroN*	24	94	87	87	60	<0.001
*iucC*	24	35	90	86	84	<0.001
*iutA*	25	35	90	84	84	<0.001
Microcin V gene	24	29	81	74	52	<0.001
*papC*	90	55	100	95	96	<0.001
*tcpC*	90	0	0	3	0	<0.001
*traT*	92	84	94	87	88	0.607
Antigen 43 gene	94	87	4	95	4	<0.001
*cdiA*	0	58	0	0	0	<0.001
Microcin H47 gene	22	29	71	48	70	<0.001
*tia*	0	74	2	0	0	<0.001
*cdtB*	0	29	2	5	4	<0.001
*senB*	69	42	2	0	4	<0.001

a Contingency table χ^2^ testing if gene is nonuniformly distributed among CC95 subgroups.

### (iv) Antibiotic resistance determinants.

None of the 200 CC95 isolates had the chromosomal mutations in *gyrA* or *parC* associated with fluoroquinolone resistance. To investigate the extent to which the presence of resistant determinants that are typically plasmid associated varied among CC95 subgroups, the analysis was restricted to the 83 Australian strains from humans for which whole-genome sequence (WGS) data were available. Preliminary analysis indicated that presence of particular resistance determinants varied in relation to both serotype and CC95 subgroup. Consequently, for statistical comparisons the subgroup A strains were split into serotypes O1:H7 and O2a:H4, and the subgroup C strains were split into serotypes O1:H7 and O25b:H4. Subgroup D and the unassigned strains were excluded due to small sample sizes. Statistical analysis of individual plasmid-borne antibiotic resistance determinants was restricted to those determinants observed 6 or more times (the number of subgroup/serotype categories).

Overall, plasmid-borne resistant determinants were uncommon among the 83 CC95 strains, with the numbers of detected determinants per strain (shown as a percentage of 83 strains in parentheses) being 0 (31.3%), 1 (25.3%), 2 (4.8%), 3 (18.1%), and ≥4 (20.5%). The number of plasmid-borne determinants varied significantly with the serotype/subgroup membership of a strain (Kruskal-Wallis test, χ^2^ = 19.6, *P* = 0.0015). Subgroup A O1:H7 and subgroup B O18:H7 strains carried, on average, less than 1 plasmid-encoded resistance determinant each, subgroup A O2a:H7, subgroup C O1:H7, and subgroup EO2a:H4 strains typically carried 2 such determinants, and subgroup C O25b:H4 strains carried more than 4 such determinants (Table 2). The full plasmid-encoded antibiotic resistance gene profiles of all CC95 study isolates are presented in Table S1.

**TABLE 2:** Frequency of plasmid-borne antibiotic resistance determinants among CC95 isolates from humans living in Canberra, Australia, with respect to their subgroup membership and serotype

Variant	% of subgroup isolates with determinant						*P* value^a^
	A, O1:H7 (*n =* 11)	A, O2:H7 (*n =* 18)	B, O18:H7 (*n =* 12)	C, O1:H7 (*n =* 18)	C, O25b:H4 (*n =* 8)	E, O2:H4 (*n =* 15)	
*aadA1*	0	57.9	0	0	50.0	0	<0.001
*bla*_TEM-1B_	9.1	52.6	41.7	5.6	62.5	46.7	0.005
*bla*_TEM-1C_	27.3	21.1	8.3	5.6	12.5	6.7	0.48
*strA*	9.1	21.1	0	16.7	12.5	26.7	0.29
*strB*	0	0	0	11.1	0	26.7	0.03
*sul1*	0	63.2	0	16.7	25.0	20.0	<0.001
*sul2*	9.1	21.1	0	16.7	12.5	26.7	0.49
*tetB*	0	0	0	88.9	25.0	0	<0.001

a Contingency table χ^2^ testing if gene is nonuniformly distributed among CC95 subgroups.

### Intrinsic extraintestinal virulence of CC95.

Phylogroup B2 strains generally are known to be highly virulent in mouse models of extraintestinal infection (24). The virulence of 58 CC95 strains isolated from Australia, France, and the United States, which included representatives of all five CC95 subgroups, was assayed using the mouse sepsis model. Consistent with their expected high virulence, 82% of strains killed all of the mice challenged with the strain. The strains’ subgroup membership explained some of the variation in ability to kill 100% of tested mice (contingency table analysis: likelihood ratio, χ^2^ = 12.27, *P* = 0.002). Killing of all tested mice was observed for all subgroup D strains (*n =* 12), 94% of subgroup B strains (*n =* 18), and 62% of subgroup A strains (*n =* 24). Strains belonging to CC95 subgroups E and C also killed 100% of mice tested, but were not included in the preceding analysis, as only a total of 6 strains from these two subgroups were tested.

### Multiplex PCR for CC95 subgroup assignment.

**FIG 5:**
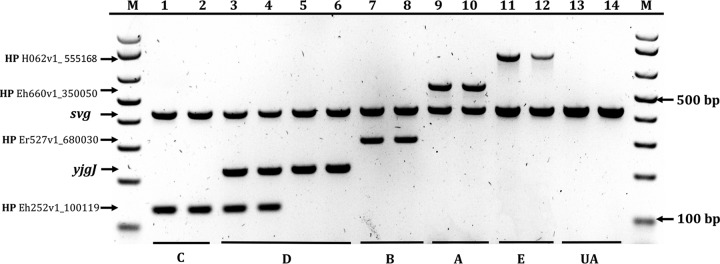
*E. coli* CC95 subgrouping multiplex PCR. The *svg* band corresponds to the CC95 control band (positive on all strains), whereas the HP H062v1_5555168, HP Eh660v1_350050, HP Er527v1_680030, *yjgJ*, and HP Eh252v1_100119 bands correspond to the amplification of subgroup E-, A-, B-, D-, and C-specific genes, respectively. UA, unassignable. Lanes: 1, H252 (O1:H7); 2, 004-008 (O25b:H4); 3, B5H8 (O1:H7); 4, B1I8 (O2a:H4) (note the amplification of the subgroup C-specific gene in addition to the subgroup D-specific gene); 5, S88 (O45a:H7); 6, 034-018 (O1:H7); 7, UTI89 (O18:H7); 8, 003-007 (O18:H7); 9, H660 (O1:H7); 10, B6H13 (O2a:H7); 11, 0032-001 (O2a:H4); 12, H062 (O2a:H4); 13, H151 (O1:H1); 14, NILS21 (O2b:H5). M, molecular size marker (1-kb Plus DNA ladder; Invitrogen).

The pangenome analysis revealed which genes were present in all members of a subgroup, but rare or absent in the other subgroups. This allowed a multiplex PCR screening tool to be developed for subgroup identification (see Materials and Methods for details and Fig. 5 for an example of the assay). Application of the multiplex method to those strains from Australia and France for which whole-genome sequence data were available showed that the method correctly assigned 92% of the strains to the appropriate subgroup (Table 3).

**TABLE 3:** Assessment of the ability of the subgroup CC95-specific genes together with serotype to correctly assign CC95 strains to their correct subgroup

Phylogeny assignment	Multiplex PCR assignment(s)	PCR serotype	No. of isolates^a^
A		O2a:H7	5
A	A	O1:H-	3
A	A	O1:H7	9
A	A	O2a:H7	16
B	B	O18:H7	19
C		O1:H7	1
C	C	O1:H7	32
C	C	O25b:H4	9
D	D	O1:H7	6
D	D	O2a:H4	2
D	D	O45:H7	2
D	D and C	O1:H7	3
D	D and C	O2a:H4	1
E	E	O2a:H4	16
Other		O1:H1	3
Other	C	O2a:H7	1
Other	A	O120:H5	1
Other	A	O2b:H5	2

a Only those strains sequenced as part of this study have been included.

### Epidemiology of CC95 subgroups.

The availability of simple PCR-based screening methods for determination of the CC95 subgroup membership of CC95 strains allowed collections of CC95 isolates from Australia, France, and the United States to be screened for the relative abundance of the CC95 subgroups. This in turn allowed an analysis of factors influencing the relative abundance of CC95 subgroups.

### (i) Distribution of CC95 subgroups in Australia.

Two large collections of *E. coli* isolates collected from human feces, urine, and blood by the Microbiology Laboratory of the Canberra Hospital, Australia, in 2002 (20) and again in 2014 and 2015 yielded 172 CC95 strains, distributed by subgroup as follows: 48% A, 18% B, 18% C, 3% D, 9% E, and 4% indeterminate. Host age and sex were known for 147 CC95 strains, in addition to the strain’s source and year of isolation. Strains were classified as having either a fecal or extraintestinal (blood or urine) source. Strains of subgroups D and E and unassigned strains were uncommon and so were pooled (Fig. 1C). Nominal logistic regression analysis revealed that the relative abundance of CC95 subgroups varied significantly with strain source, year of isolation, and host sex, but not host age (isolate source, *P* = 0.04; year of isolation, *P* < 0.001; host sex, *P* = 0.01; year of isolation-host sex interaction, *P* = 0.003). (Backwards elimination was used to eliminate nonsignificant terms, and only statistically significant terms are presented.)

The underlying prevalence values were inspected to determine the basis for these results. For the 2002 isolates, subgroup B strains were isolated more frequently from extraintestinal infection and from males than females. For the 2015 isolates, subgroup A strains were more frequent among fecal isolates than extraintestinal isolates, subgroup B strains were confined to females, and subgroup C strains were more common among females than males. In comparison with the 2002 isolates, the 2015 isolates had a lower frequency of subgroup B and a higher frequency of subgroup C, the latter reflecting an increase in the frequency of both the O1:H7 and O25b:H4 serotypes.

### (ii) Distribution of CC95 subgroups in France.

Four collections of *E. coli* isolates from France (two of blood isolates and two of fecal isolates) were screened for CC95 strains, and the subgroup membership of the identified CC95 strains was determined. The blood isolates were collected in 2005 (25) and in 2005 to 2007 (13), while the fecal isolates were collected in 2000 (26) and 2010 (27). The 208 CC95 strains detected in these collections were distributed by subgroup as follows: 4% A, 12% B, 4% C, 69% D, and 9% E, and 1 strain could not be assigned to a subgroup.

Although the two sets of blood isolates were collected at similar times, they were taken from either adults or children, while the two sets of fecal isolates were collected 10 years apart. Thus, sample sizes were insufficient to permit an analysis that incorporated the effects of isolate source, host age, and year. However, a significant difference in the relative abundance of CC95 subgroups was detected when comparing the CC95 fecal and blood isolates (contingency table analysis: likelihood ratio, χ^2^ = 12.46, *P* = 0.01). Among the 174 CC95 blood isolates, 4% were subgroup A, 10% B, 3% C, 74% D, and 16% E, and 1 isolate was unassigned. In contrast, among the 33 CC95 fecal isolates, 6% were A, 26% B, 12% C, 47% D, and 9% E. Whereas the CC95 blood strains belonged overwhelmingly to subgroup D, the fecal strains, although also most likely to belong to subgroup D, also frequently belonged subgroup B.

In these collections, host age and sex were known for 147 CC95 strains isolated from blood. The relative abundance of CC95 subgroups varied with host sex but not with host age (nominal logistic regression: age, χ^2^ = 5.06, *P* = 0.28; sex, χ^2^ = 17.55, *P* = 0.0015; age-sex interaction, χ^2^ = 6.15, *P* = 0.188). Among the 114 CC95 blood isolates recovered from females, 3% were A, 6% B, 3% C, 77% D, and 11% E. In contrast, among the 33 blood isolates from males, 6% were A, 30% B, 0% C, 55% D, and 9% E. Although subgroup D isolates were the most likely subgroup to be recovered from females, subgroup D isolates were less likely to be observed among blood isolates from males and subgroup B isolates were more frequent.

### (iii) Distribution of CC95 subgroups in the United States.

Seven collections of *E. coli* strains from the United States acquired between 1981 and 2015 from human feces, blood, urine, and other extraintestinal sites and diverse localities (22, 28–32), were screened for CC95, and the subgroup membership of the identified CC95 strains was determined. The 146 detected CC95 strains were distributed by subgroup as follows: 65% A, 27% B, 3% D, and 4% E. None represented subgroup C.

A comparison of CC95 strains from blood and urine identified a significant source effect on the relative abundance of CC95 subgroups (contingency table analysis: likelihood ratio, χ^2^ = 6.46, *P* > χ^2^ = 0.039). The blood isolates belonged mainly to subgroup A (70%), with only a small minority from subgroup B (13%) or other backgrounds (17%). In contrast, although the urine isolates likewise belonged mainly to subgroup A (62%), 33% belonged to subgroup B, and only 5% represented other backgrounds.

The 104 urine isolates were collected over 27 years and exhibited a significant change in relative subgroup abundance over this period (nominal logistic regression: year of isolation, χ^2^ = 5.06, *P* = 0.28; sex, χ^2^ = 17.55, *P* = 0.0015; age-sex interaction, χ^2^ = 15.09, *P* < 0.001). Specifically, subgroup A declined in relative frequency, with a concomitant increase in the other subgroups.

### (iv) Among-country subgroup frequency comparisons.

The relative abundance of CC95 subgroups varies with the strain’s source of isolation (blood, urine, or feces) (Fig. 1C), and the available CC95 data are quite biased among countries with respect to isolate source. Therefore, comparisons of the relative abundance of different subgroups among CC95 strains from Australia, France, and the United States were restricted to strains from the same sources.

In these specimen-type-stratified comparisons, among the urine isolates, those from the United States were predominantly from subgroups A and B, while those from Australia were predominantly from subgroups A and C (Table 4). Among the blood isolates, those from France were predominantly from subgroup D, whereas none of those from Australia were from subgroup D. Among the fecal isolates, those from France were mostly from subgroup D, but subgroup B isolates were also quite common, whereas those from Australia were mostly from subgroup A.

**TABLE 4:** Relative abundance of CC95 subgroups isolated from the same source in different countries

Source	Country	*n*	% of isolates of subgroup:					*P* value^a^
			A	B	C	D	E	
Urine	Australia	41	41	14	30	0	11	<0.001
	United States	99	66	29	0	2	3	
Blood	Australia	23	33	11	33	0	14	<0.001
	France	173	4	10	3	74	9	
Feces	Australia	27	64	11	14	4	7	<0.001
	France	34	6	26	12	47	9	

a Contingency table χ^2^ testing if CC95 subgroups are nonuniformly distributed with respect to source.

Virulence factor data obtained through PCR screening were available for many of the non-Australian CC95 strains as well as the Australian strains. However, given the very nonrandom distribution of CC95 subgroups among countries, there were insufficient sample sizes to compare strains belonging to all subgroups among all countries. Therefore, a comparison of the distributions of virulence factors was restricted to CC95 subgroup B (Table 5). These analyses revealed that the virulence trait profile of subgroup B CC95 isolates varied with the country of origin.

**TABLE 5:** Frequency of specific virulence traits in CC95 subgroup B isolates from different countries

Virulence trait	% of isolates positive from:			*P* value^a^
	Australia (*n =* 12)	France (*n =* 25)	United States (*n =* 21)	
*papC*	67	4	71	<0.001
*iroN*	67	100	95	0.005
*iut*	33	80	5	<0.001
*cnf1*	58	8	95	<0.001

a Contingency table χ^2^ testing if gene is nonuniformly distributed among CC95 subgroups.

Of the observed geographic differences in virulence trait profiles, the most striking related to the *pap* operon. Specifically, the *pap* operon was absent in most isolates from France but present in most isolates from Australia and the United States, whereas among *pap*-positive isolates, all of those from France and the United States had *papG* allele III, while those from Australia had *papG* allele II. As for other virulence factors, the salmochelin locus (*iroN*) was less frequent among isolates from Australia than those from France or the United States, the aerobactin locus (*iut*) was common among isolates from France, but absent from most isolates from the United States, and cytotoxic necrotizing factor 1 (*cnf1*) was uncommon among isolates from France, but present in almost all isolates from the United States.

## DISCUSSION

*E. coli* CC95 has a cosmopolitan distribution, as it has been recovered from every continent, including Antarctica. This clonal complex has a narrow host range—one seemingly restricted to humans and human-associated birds and mammals, such as commercial poultry and companion animals. Although data are limited, the available evidence indicates that CC95 strains are capable of persisting in the human gut for extended periods, as has been observed for other B2 strains, as residence times of 2 to 4 years have been observed (33). Strains of the complex are significant extraintestinal pathogens of humans and globally appear to account for about 17% of infections caused by *E. coli*.

The pangenome of CC95 was estimated to be in excess of 17,000 genes, a value similar to that found in a collection of 20 strains representative of *E. coli* as a whole (34). The distribution of these genes among genomes was also very similar to that reported by Touchon et al. (34); about 21% of genes were present in >95% of the isolates, while 68% were present in <15% of isolates.

The existence of genetic substructure in CC95 has long been recognized (35, 36), and based on variation in the core genome, it appears that the great majority of ST95 isolates belong to one of five subgroups. The subgroups are nonrandomly distributed geographically, with subgroup D strains being encountered more commonly in Europe than in Australia or North America. Subgroup C strains with an O1:H7 serotype may be endemic to Australia, as they have not been observed in other parts of the world. The distribution of extraintestinal virulence traits also varies among subgroups and likewise in relation to geographic origin. These geographic differences extend to different alleles of a particular gene, as noted for *papG* allele III, which is far more likely to be observed among CC95 isolates from the United States than those from Australia.

The frequency of CC95 in human populations would be expected to change if the frequency of phylogroup B2 strains changed, as has been the case in France over the past 30 years (27). In the Australian *E. coli* collections, however, there was no change in the frequency of phylogroup B2 strains between 2002 and 2014, irrespective of a strain’s origin (feces, urine, or blood) (Fig. 1A). Similarly, there was no change in the frequency of CC95 strains in the Australian collections with respect to either sample date or a strain’s origin (Fig. 1B). Similarly, Day et al. (37), in examining the clonal composition of *E. coli* strains causing bacteremia in the United Kingdom and Ireland between 2001 and 2010, found that CC95 strains were responsible for 11.3% of bacteremia cases overall, with no real change in frequency over time.

In stark contrast to the apparent temporal stability within *E. coli* of the relative abundance of CC95 at the clonal complex level is the variability within CC95 of the relative abundance of the various CC95 subgroups between 2002 and 2104 in Australia (Fig. 1C) and between 2011 and 2015 in the United States. However, the natures of the changes on the two continents were different. In Australia, subgroup B isolates decline in frequency, while subgroup C isolates became more common, whereas in the United States, subgroup A declined in frequency and the frequency of subgroup B was unchanged.

The substructure that exists in CC95 is epidemiologically relevant as, depending on geographic location, strains of some subgroups are more likely to cause septicemia than urinary tract infection or are more likely to be recovered from feces than from extraintestinal sites. Members of some subgroups also appear to less likely to be recovered from males or more likely to be recovered from extraintestinal sites in females. Additional support for the epidemiological relevance of the subgroups comes from results from the mouse sepsis model, which reflects a strain’s extraintestinal virulence. These results indicate that although most CC95 strains are highly virulent, some are not, and that some of this variation in virulence is explained by a strain’s subgroup membership.

Collectively, the available evidence indicates that although strains belonging to CC95 may be cosmopolitan, human movement patterns have been insufficient to homogenize the distribution of the CC95 subgroups. Rather, the manner in which CC95 strains evolve appears to vary both spatially and temporally. The observation that the relative frequency of CC95 subgroups at a single locality has changed over time indicates that the relative fitness of the subgroups has changed, although whether such changes are relative to other members of the clonal complex or to other strains of *E. coli* is unknown. Also unknown is the extent to which these apparent changes in relative fitness are a consequence of direct competition between members of the different subgroups and of how the different subgroups respond to changes in the host/external environment. Stochastic effects undoubtedly also play a role, as might be the case for the dominance of *papG* allele III among subgroup B strains in North America.

The ST131 clonal complex (CC131) is another very common human-associated *E. coli* lineage. However, few similarities are apparent between the evolution of CC131 and CC95. CC131 is a genetically more diverse clonal complex than CC95, but much of the success of CC131 is due to the evolution and spread of two very closely related, virtually monomorphic, fluoroquinolone-resistant lineages with an O25b:H4 serotype, known as *H*30R/C1 and *H*30Rx/C2 (9, 10). These lineages were virtually unknown prior to the turn of the century but have now spread worldwide and are currently the *E. coli* lineages most likely to be responsible for extraintestinal infection (38). Although a number of hypotheses have been proposed to explain the success of these ST131 lineages, such as enhanced virulence and antibiotic resistance, none have strong empirical support.

Collectively, the evidence indicates that although CC95 may be a temporally stable pandemic lineage of *E. coli* that is often responsible for extraintestinal disease in humans, its evolution bears little resemblance to that of other pandemic pathogenic lineages, such as *Salmonella enterica* serovar Typhi, *Shigella sonnei*, or even *E. coli* CC131. Rather, the evolution of CC95 is shaped by local conditions operating on at least a continental scale and over relatively short time frames. Moreover, the differences among the various CC95 subgroups are epidemiologically relevant. The analytical approaches that have been used so successfully to investigate the evolution and spread of other human pathogens, such as *Yersinia pestis* and *Salmonella enterica* serovar Agona (39), are unlikely to be as successful for CC95 strains, given the extent of spatial and temporal variation observed in this complex.

The pangenome analysis revealed that the genetic structure of *Escherichia coli* is fractal in nature. At the species level, *E. coli* exhibits considerable genetic structure (phylogroups), as discerned by examining the phylogenetic relationships among strains based on genes of the core genome. This substructure is reflected also in the similarity analyses based on the species’ variable gene content (40). The distribution of genes is such that a minority of genes of the pangenome are present in all strains, while most genes in the pangenome are present in just one or a few strains. Notably, the same patterns are observed among the strains within a single clonal complex. CC95 exhibits substructure in its core genome, and this substructure is reflected in the variable genome of CC95, with most genes present in few isolates and a minority of genes present in all isolates. The results of this analysis of CC95 pangenome and of the analysis by Touchon et al. (34) of the *E. coli* pangenome cannot be compared directly due to differences in methodology. However, the size of the pangenome of CC95 is certainly close to that of the species as a whole. It is remarkable that the same pangenome can reflect the core genome structure observed for both the whole species and a clonal complex within the species. Further studies are required to elucidate the evolutionary processes that might lead to the observed patterns.

## MATERIALS AND METHODS

### Determining the CC95 pangenome.

The nature of the pangenome of CC95 was investigated using WGS data for 200 CC95 strains. These included a collection of strains from Australia (*n* = 114) and France (*n =* 17) for which WGS data (Roche 454 GS FLX+ system, Illumina MiSeq, and Illumina HiSeq2000) were available plus a collection of CC95 strains (*n* = 66) as identified by Deshpande et al. (55) for which WGS data were available from NCBI, as well as three CC95 reference strains (APECO1, S88, and UTI89) (Table S1). The assemblies and annotations for all non-NCBI strains are available in Enterobase (http://enterobase.warwick.ac.uk/).

The assembled genomes were annotated using Prokka (41), and ROARY (42) was used for the pangenome analysis. The Harvest suite of tools (43) was used to align the strains and visualize the inferred phylogenetic relationships of these 200 CC95 strains. As expected, the phylogeny revealed significant structure within CC95, and the observed clustering largely correlated with a strain’s serotype (Fig. 3).

### CC95 *in silico* characterization.

Where required, the 200 CC95 isolates were assigned to sequence types (STs) using the University of Warwick multilocus sequence type (MLST) scheme (http://enterobase.warwick.ac.uk/). The Center for Genomic Epidemiology (CGE) website (http://www.genomicepidemiology.org) was used to characterize the strains, using the ResFinder (44) and SeroTypeFinder (45) tools. The presence of mutations in *gyrA* and *parC* associated with fluoroquinolone resistance was determined using RGI (https://card.mcmaster.ca/analyze/rgi) (46). The presence of 40 virulence factor genes (Table S1) was determined using a BLAST search (CLC Genomics Workbench v9).

### Subgroup detection using PCR-based typing.

SCOARY (47) was used to identify genes found be present in virtually all members of a subgroup and rare or absent in all other subgroups. The resulting gene sets were then examined for genes likely to be suitable for PCR targeting.

A hypothetical protein-coding gene was found to be present in all subgroup A strains and absent in all but three other CC95 strains (B525, E8766, and E9345). A different hypothetical protein-coding gene was found to be present in all subgroup C strains, although this gene was also present in three subgroup D strains. A glycosyltransferase group 2 family protein-coding gene was unique to subgroup B strains. Subgroup D strains were defined by the presence of *yjgJ*, a putative transcriptional regulator-coding gene. Subgroup E strains were defined by the *E. coli* restriction-modification enzyme type I M subunit (*hsdM*) gene. The nucleotide sequences for these genes are presented in Table S2 in the supplemental material.

10.1128/mSphere.00168-17.2 TABLE S2  Nucleotide sequences of genes found to be unique to one of the five CC95 subgroups. Download TABLE S2, DOCX file, 0.1 MB. Copyright © 2017 Gordon et al. 2017 Gordon et al. This content is distributed under the terms of the Creative Commons Attribution 4.0 International license.

Primers were designed to specifically target each of these genes, such that fragments of each unique gene could be amplified in a single multiplex PCR (see Table S3 in the supplemental material). Bidet et al. (48) developed a CC95-specific PCR based on the presence of an open reading frame (ORF) of unknown function that they designated *svg*. The *svg* primers were included in the primer pool; *svg* serves as both a positive control for the subgrouping multiplex PCR and an additional verification of an isolate’s identity as CC95. All PCRs used a 20-µl volume containing 4 µl of 5× MyTaq Red buffer (supplied with MyTaq *Taq* polymerase [Bioline]), 2 U of *Taq* polymerase, 1.2 µl of DNA (at approximately 100 ng/µl), and 20 pmol of each primer. The PCR conditions were denaturation for 4 min at 94°C, 30 cycles of 5 s at 94°C and 20 s at 59°C, and a final extension step of 5 min at 72°C.

10.1128/mSphere.00168-17.3 TABLE S3  Primer sequences and characteristics used for the classification of CC95 strains into five subgroups. Download TABLE S3, DOCX file, 0.1 MB. Copyright © 2017 Gordon et al. 2017 Gordon et al. This content is distributed under the terms of the Creative Commons Attribution 4.0 International license.

### Distribution of CC95 and CC95 subgroups.

A number of existing *E. coli* strain collections acquired for a variety of reasons and over a number of years from hosts living in Australia, France, and the United States were screened to determine which strains in the collections belonged to CC95. Additional details of these collections are presented in the Results section. The phylogroup membership of these strains had been determined previously. CC95 identity was determined by either multilocus sequencing typing or PCR-based screening using one or more of the published CC95 detection methods (7, 48, 49). The methods used for PCR-based virulence factor screening are described in the references cited for each of the strain collections used in this study.

All CC95 strains identified in the various strain collections were then screened using the CC95 subgroup multiplex PCR, together with PCR-based O typing (50) and H typing for H7 (51) and H4 (52). Any strain yielding an *svg* product together with a subgroup C and subgroup D PCR product was considered a subgroup D strain (Fig. 5).

### Intrinsic extraintestinal virulence.

The virulence of CC95 strains was tested in a mouse model of sepsis following neck subcutaneous inoculation of 2 × 10^8^ bacteria as described by Johnson et al. (24). Briefly, 10 outbred female Swiss mice were inoculated per strain, and death was monitored up to 7 days after inoculation. In each experiment, two *E. coli* control strains were systematically tested: K-12 strain MG1655, which does not kill mice, and strain CFT073, which kills 100% of inoculated mice. Experiments conducted in France (27 strains belonging to all subgroups) followed the European and national regulations for housing and care of laboratory animals after pertinent review and approval by the Bioethics Committee at Santiago de Compostela University and by the French Veterinary Services (certificate no. A 75-18-05). Experiments conducted in Minnesota (31 strains belonging to subgroups A, B, and D) followed federal regulations for ethical care and use of laboratory animals, with approval by the local Institutional Animal Care and Use Committee (protocol 120603).

### Statistical analysis.

Factors influencing the relative frequency of CC95 subgroups were investigated using either contingency table analysis or nominal logistic regression where appropriate. For analyses involving multiple factors, nonsignificant terms were dropped using backwards elimination, and only significant terms were reported.

### Accession number(s).

The raw sequence read files have been deposited in NCBI and are associated with BioProject PRJNA385370, accession no. SRX2786117 to SRX2786219.

## References

[1] Achtman M 2012 Insights from genomic comparisons of genetically monomorphic bacterial pathogens. Philos Trans R Soc Lond B Biol Sci 367:860–867. doi:10.1098/rstb.2011.0303. 22312053 PMC3267118

[2] Bart MJ, van Gent M, van der Heide HG, Boekhorst J, Hermans P, Parkhill J, Mooi FR 2010 Comparative genomics of prevaccination and modern Bordetella pertussis strains. BMC Genomics 11:627. doi:10.1186/1471-2164-11-627. 21070624 PMC3018138

[3] Cui Y, Yu C, Yan Y, Li D, Li Y, Jombart T, Weinert LA, Wang Z, Guo Z, Xu L, Zhang Y, Zheng H, Qin N, Xiao X, Wu M, Wang X, Zhou D, Qi Z, Du Z, Wu H, Yang X, Cao H, Wang H, Wang J, Yao S, Rakin A, Li Y, Falush D, Balloux F, Achtman M, Song Y, Wang J, Yang R 2013 Historical variations in mutation rate in an epidemic pathogen, Yersinia pestis. Proc Natl Acad Sci U S A 110:577–582. doi:10.1073/pnas.1205750110. 23271803 PMC3545753

[4] Holt KE, Parkhill J, Mazzoni CJ, Roumagnac P, Weill FX, Goodhead I, Rance R, Baker S, Maskell DJ, Wain J, Dolecek C, Achtman M, Dougan G 2008 High-throughput sequencing provides insights into genome variation and evolution in Salmonella typhi. Nat Genet 40:987–993. doi:10.1038/ng.195. 18660809 PMC2652037

[5] Schuenemann VJ, Singh P, Mendum TA, Krause-Kyora B, Jäger G, Bos KI, Herbig A, Economou C, Benjak A, Busso P, Nebel A, Boldsen JL, Kjellström A, Wu H, Stewart GR, Taylor GM, Bauer P, Lee OY, Wu HH, Minnikin DE, Besra GS, Tucker K, Roffey S, Sow SO, Cole ST, Nieselt K, Krause J 2013 Genome-wide comparison of medieval and modern Mycobacterium leprae. Science 341:179–183. doi:10.1126/science.1238286. 23765279

[6] Zhou Z, McCann A, Weill FX, Blin C, Nair S, Wain J, Dougan G, Achtman M 2014 Transient Darwinian selection in Salmonella enterica serovar Paratyphi A during 450 years of global spread of enteric fever. Proc Natl Acad Sci U S A 111:12199–12204. doi:10.1073/pnas.1411012111. 25092320 PMC4143038

[7] Clermont O, Christenson JK, Daubié AS, Gordon DM, Denamur E 2014 Development of an allele-specific PCR for Escherichia coli B2 sub-typing, a rapid and easy to perform substitute of multilocus sequence typing. J Microbiol Methods 101:24–27. doi:10.1016/j.mimet.2014.03.008. 24685601

[8] Riley LW 2014 Pandemic lineages of extraintestinal pathogenic Escherichia coli. Clin Microbiol Infect 20:380–390. doi:10.1111/1469-0691.12646. 24766445

[9] Ben Zakour NL, Alsheikh-Hussain AS, Ashcroft MM, Khanh Nhu T, Roberts LW, Stanton-Cook M, Schembri MA, Beatson SA 2016 Sequential acquisition of virulence and fluoroquinolone resistance has shaped the evolution of Escherichia coli ST131. mBio 7:e00347-16. doi:10.1128/mBio.00347-16. 27118589 PMC4850260

[10] Stoesser N, Sheppard AE, Pankhurst L, De Maio N, Moore CE, Sebra R, Turner P, Anson LW, Kasarskis A, Batty EM, Kos V, Wilson DJ, Phetsouvanh R, Wyllie D, Sokurenko E, Manges AR, Johnson TJ, Price LB, Peto TE, Johnson JR, Didelot X, Walker AS, Crook DW, Modernizing Medical Microbiology Informatics Group (MMMIG) 2016 Evolutionary history of the global emergence of the Escherichia coli epidemic clone ST131. mBio 7:e02162. doi:10.1128/mBio.02162-15. 27006459 PMC4807372

[11] Dias RC, Marangoni DV, Smith SP, Alves EM, Pellegrino FL, Riley LW, Moreira BM 2009 Clonal composition of Escherichia coli causing community-acquired urinary tract infections in the State of Rio de Janeiro, Brazil. Microb Drug Resist 15:303–308. doi:10.1089/mdr.2009.0067. 19857137 PMC3145954

[12] Banerjee R, Johnston B, Lohse C, Chattopadhyay S, Tchesnokova V, Sokurenko EV, Johnson JR 2013 The clonal distribution and diversity of extraintestinal Escherichia coli isolates vary according to patient characteristics. Antimicrob Agents Chemother 57:5912–5917. doi:10.1128/AAC.01065-13. 24041881 PMC3837911

[13] Burdet C, Clermont O, Bonacorsi S, Laouénan C, Bingen E, Aujard Y, Mentré F, Lefort A, Denamur E, COLIBAFI Group 2014 Escherichia coli bacteremia in children: age and portal of entry are the main predictors of severity. Pediatr Infect Dis J 33:872–879. doi:10.1097/INF.0000000000000309. 25222308

[14] Alghoribi MF, Gibreel TM, Farnham G, Al Johani SM, Balkhy HH, Upton M 2015 Antibiotic-resistant ST38, ST131 and ST405 strains are the leading uropathogenic Escherichia coli clones in Riyadh, Saudi Arabia. J Antimicrob Chemother 70:2757–2762. doi:10.1093/jac/dkv188. 26183183

[15] Basmaci R, Bonacorsi S, Bidet P, Biran V, Aujard Y, Bingen E, Béchet S, Cohen R, Levy C 2015 Escherichia coli meningitis features in 325 children from 2001 to 2013 in France. Clin Infect Dis 61:779–786. doi:10.1093/cid/civ367. 25944342

[16] Yun KW, Kim DS, Kim W, Lim IS 2015 Molecular typing of uropathogenic Escherichia coli isolated from Korean children with urinary tract infection. Korean J Pediatr 58:20–27. doi:10.3345/kjp.2015.58.1.20. 25729395 PMC4342777

[17] Wang S, Zhao SY, Xiao SZ, Gu FF, Liu QZ, Tang J, Guo XK, Ni YX, Han LZ 2016 Antimicrobial resistance and molecular epidemiology of Escherichia coli causing bloodstream infections in three hospitals in Shanghai, China. PLoS One 11:e0147740. doi:10.1371/journal.pone.0147740. 26824702 PMC4733056

[18] Blyton MD, Pi H, Vangchhia B, Abraham S, Trott DJ, Johnson JR, Gordon DM 2015 Genetic structure and antimicrobial resistance of Escherichia coli and cryptic clades in birds with diverse human associations. Appl Environ Microbiol 81:5123–5133. doi:10.1128/AEM.00861-15. 26002899 PMC4495204

[19] Gordon DM, Cowling A 2003 The distribution and genetic structure of Escherichia coli in Australian vertebrates: host and geographic effects. Microbiology 149:3575–3586. doi:10.1099/mic.0.26486-0. 14663089

[20] Gordon DM, Stern SE, Collignon PJ 2005 Influence of the age and sex of human hosts on the distribution of Escherichia coli ECOR groups and virulence traits. Microbiology 151:15–23. doi:10.1099/mic.0.27425-0. 15632421

[21] Weissman SJ, Johnson JR, Tchesnokova V, Billig M, Dykhuizen D, Riddell K, Rogers P, Qin X, Butler-Wu S, Cookson BT, Fang FC, Scholes D, Chattopadhyay S, Sokurenko E 2012 High-resolution two-locus clonal typing of extraintestinal pathogenic Escherichia coli. Appl Environ Microbiol 78:1353–1360. doi:10.1128/AEM.06663-11. 22226951 PMC3294456

[22] Johnson JR, Porter S, Johnston B, Kuskowski MA, Spurbeck RR, Mobley HL, Williamson DA 2015 Host characteristics and bacterial traits predict experimental virulence for Escherichia coli bloodstream isolates from patients with urosepsis. Open Forum Infect Dis 2:ofv083. doi:10.1093/ofid/ofv083. 26199950 PMC4504731

[23] Johnson JR, Russo TA 15 11 2004 Molecular epidemiology of extraintestinal pathogenic Escherichia coli. EcoSal Plus 2004. doi:10.1128/ecosalplus.8.6.1.4. 26443356

[24] Johnson JR, Clermont O, Menard M, Kuskowski MA, Picard B, Denamur E 2006 Experimental mouse lethality of Escherichia coli isolates, in relation to accessory traits, phylogenetic group, and ecological source. J Infect Dis 194:1141–1150. doi:10.1086/507305. 16991090

[25] Lefort A, Panhard X, Clermont O, Woerther PL, Branger C, Mentré F, Fantin B, Wolff M, Denamur E, COLIBAFI Group 2011 Host factors and portal of entry outweigh bacterial determinants to predict the severity of Escherichia coli bacteremia. J Clin Microbiol 49:777–783. doi:10.1128/JCM.01902-10. 21177892 PMC3067752

[26] Skurnik D, Clermont O, Guillard T, Launay A, Danilchanka O, Pons S, Diancourt L, Lebreton F, Kadlec K, Roux D, Jiang D, Dion S, Aschard H, Denamur M, Cywes-Bentley C, Schwarz S, Tenaillon O, Andremont A, Picard B, Mekalanos J, Brisse S, Denamur E 2016 Emergence of antimicrobial-resistant Escherichia coli of animal origin spreading in humans. Mol Biol Evol 33:898–914. doi:10.1093/molbev/msv280. 26613786 PMC5013867

[27] Massot M, Daubié AS, Clermont O, Jauréguy F, Couffignal C, Dahbi G, Mora A, Blanco J, Branger C, Mentré F, Eddi A, Picard B, Denamur E, Coliville Group 2016 Phylogenetic, virulence and antibiotic resistance characteristics of commensal strain populations of Escherichia coli from community subjects in the Paris area in 2010 and evolution over 30 years. Microbiology 162:642–650. doi:10.1099/mic.0.000242. 26822436 PMC6365622

[28] Sannes MR, Kuskowski MA, Johnson JR 2004 Antimicrobial resistance of Escherichia coli strains isolated from urine of women with cystitis or pyelonephritis and feces of dogs and healthy humans. J Am Vet Med Assoc 225:368–373. doi:10.2460/javma.2004.225.368. 15328711

[29] Banerjee R, Johnston B, Lohse C, Porter SB, Clabots C, Johnson JR 2013 Escherichia coli sequence type 131 is a dominant, antimicrobial-resistant clonal group associated with healthcare and elderly hosts. Infect Control Hosp Epidemiol 34:361–369. doi:10.1086/669865. 23466908 PMC3916146

[30] Colpan A, Johnston B, Porter S, Clabots C, Anway R, Thao L, Kuskowski MA, Tchesnokova V, Sokurenko EV, Johnson JR, VICTORY Investigators 2013 Escherichia coli sequence type 131 (ST131) subclone H30 as an emergent multidrug-resistant pathogen among US veterans. Clin Infect Dis 57:1256–1265. doi:10.1093/cid/cit503. 23926176 PMC3792724

[31] Drawz SM, Porter S, Kuskowski MA, Johnston B, Clabots C, Kline S, Ferrieri P, Johnson JR 2015 Variation in resistance traits, phylogenetic backgrounds, and virulence genotypes among Escherichia coli clinical isolates from adjacent hospital campuses serving distinct patient populations. Antimicrob Agents Chemother 59:5331–5339. doi:10.1128/AAC.00048-15. 26100703 PMC4538515

[32] Drekonja DM, Kuskowski MA, Anway R, Johnston BD, Johnson JR 2016 The niche for Escherichia coli sequence type 131 among veterans: urinary tract abnormalities and long-term care facilities. Open Forum Infect Dis 3:ofw138. doi:10.1093/ofid/ofw138. 27703999 PMC5047397

[33] Anantham S 2014 Analysis of persistent and antibiotic resistant commensal Escherichia coli from healthy adults. PhD thesis University of Sydney, Sydney, Australia.

[34] Touchon M, Hoede C, Tenaillon O, Barbe V, Baeriswyl S, Bidet P, Bingen E, Bonacorsi S, Bouchier C, Bouvet O, Calteau A, Chiapello H, Clermont O, Cruveiller S, Danchin A, Diard M, Dossat C, Karoui ME, Frapy E, Garry L, Ghigo JM, Gilles AM, Johnson J, Le Bouguénec C, Lescat M, Mangenot S, Martinez-Jéhanne V, Matic I, Nassif X, Oztas S, Petit MA, Pichon C, Rouy Z, Ruf CS, Schneider D, Tourret J, Vacherie B, Vallenet D, Médigue C, Rocha EP, Denamur E 2009 Organised genome dynamics in the Escherichia coli species results in highly diverse adaptive paths. PLoS Genet 5:e1000344. doi:10.1371/journal.pgen.1000344. 19165319 PMC2617782

[35] Achtman M, Mercer A, Kusecek B, Pohl A, Heuzenroeder M, Aaronson W, Sutton A, Silver RP 1983 Six widespread bacterial clones among Escherichia coli K1 isolates. Infect Immun 39:315–335. 6218094 10.1128/iai.39.1.315-335.1983 PMC347943

[36] Bidet P, Mahjoub-Messai F, Blanco J, Blanco J, Dehem M, Aujard Y, Bingen E, Bonacorsi S 2007 Combined multilocus sequence typing and O serogrouping distinguishes Escherichia coli subtypes associated with infant urosepsis and/or meningitis. J Infect Dis 196:297–303. doi:10.1086/518897. 17570118

[37] Day MJ, Doumith M, Abernethy J, Hope R, Reynolds R, Wain J, Livermore DM, Woodford N 2016 Population structure of Escherichia coli causing bacteraemia in the UK and Ireland between 2001 and 2010. J Antimicrob Chemother 71:2139–2142. doi:10.1093/jac/dkw145. 27150395 PMC4954928

[38] Banerjee R, Johnson JR 2014 A new clone sweeps clean: the enigmatic emergence of Escherichia coli sequence type 131. Antimicrob Agents Chemother 58:4997–5004. doi:10.1128/AAC.02824-14. 24867985 PMC4135879

[39] Zhou Z, McCann A, Litrup E, Murphy R, Cormican M, Fanning S, Brown D, Guttman DS, Brisse S, Achtman M 2013 Neutral genomic microevolution of a recently emerged pathogen, Salmonella enterica serovar Agona. PLoS Genet 9:e1003471. doi:10.1371/journal.pgen.1003471. 23637636 PMC3630104

[40] Kaas RS, Friis C, Ussery DW, Aarestrup FM 2012 Estimating variation within the genes and inferring the phylogeny of 186 sequenced diverse Escherichia coli genomes. BMC Genomics 13:577. doi:10.1186/1471-2164-13-577. 23114024 PMC3575317

[41] Seemann T 2014 Prokka: rapid prokaryotic genome annotation. Bioinformatics 30:2068–2069. doi:10.1093/bioinformatics/btu153. 24642063

[42] Page AJ, Cummins CA, Hunt M, Wong VK, Reuter S, Holden MT, Fookes M, Falush D, Keane JA, Parkhill J 2015 Roary: rapid large-scale prokaryote pan genome analysis. Bioinformatics 31:3691–3693. doi:10.1093/bioinformatics/btv421. 26198102 PMC4817141

[43] Treangen TJ, Ondov BD, Koren S, Phillippy AM 2014 The Harvest suite for rapid core-genome alignment and visualization of thousands of intraspecific microbial genomes. Genome Biol 15:524. doi:10.1186/PREACCEPT-2573980311437212. 25410596 PMC4262987

[44] Zankari E, Hasman H, Cosentino S, Vestergaard M, Rasmussen S, Lund O, Aarestrup FM, Larsen MV 2012 Identification of acquired antimicrobial resistance genes. J Antimicrob Chemother 67:2640–2644. doi:10.1093/jac/dks261. 22782487 PMC3468078

[45] Joensen KG, Tetzschner AM, Iguchi A, Aarestrup FM, Scheutz F 2015 Rapid and easy in silico serotyping of Escherichia coli isolates by use of whole-genome sequencing data. J Clin Microbiol 53:2410–2426. doi:10.1128/JCM.00008-15. 25972421 PMC4508402

[46] Jia B, Raphenya AR, Alcock B, Waglechner N, Guo P, Tsang KK, Lago BA, Dave BM, Pereira S, Sharma AN, Doshi S, Courtot M, Lo R, Williams LE, Frye JG, Elsayegh T, Sardar D, Westman EL, Pawlowski AC, Johnson TA, Brinkman FS, Wright GD, McArthur AG 2017 CARD 2017: expansion and model-centric curation of the comprehensive antibiotic resistance database. Nucleic Acids Res 45:D566–D573. doi:10.1093/nar/gkw1004. 27789705 PMC5210516

[47] Brynildsrud O, Bohlin J, Scheffer L, Eldholm V 2016 Rapid scoring of genes in microbial pan-genome-wide association studies with Scoary. Genome Biol 17:238. doi:10.1186/s13059-016-1108-8. 27887642 PMC5124306

[48] Bidet P, Metais A, Mahjoub-Messai F, Durand L, Dehem M, Aujard Y, Bingen E, Nassif X, Bonacorsi S 2007 Detection and identification by PCR of a highly virulent phylogenetic subgroup among extraintestinal pathogenic Escherichia coli B2 strains. Appl Environ Microbiol 73:2373–2377. doi:10.1128/AEM.02341-06. 17293507 PMC1855671

[49] Doumith M, Day M, Ciesielczuk H, Hope R, Underwood A, Reynolds R, Wain J, Livermore DM, Woodford N 2015 Rapid identification of major Escherichia coli sequence types causing urinary tract and bloodstream infections. J Clin Microbiol 53:160–166. doi:10.1128/JCM.02562-14. 25355761 PMC4290915

[50] Clermont O, Johnson JR, Menard M, Denamur E 2007 Determination of Escherichia coli O types by allele-specific polymerase chain reaction: application to the O types involved in human septicemia. Diagn Microbiol Infect Dis 57:129–136. doi:10.1016/j.diagmicrobio.2006.08.007. 17020797

[51] Johnson JR, Stell AL 2001 PCR for specific detection of H7 flagellar variant of fliC among extraintestinal pathogenic Escherichia coli. J Clin Microbiol 39:3712–3717. doi:10.1128/JCM.39.10.3712-3717.2001. 11574599 PMC88415

[52] Pritchard L, Holden NJ, Bielaszewska M, Karch H, Toth IK 2012 Alignment-free design of highly discriminatory diagnostic primer sets for Escherichia coli O104:H4 outbreak strains. PLoS One 7:e34498. doi:10.1371/journal.pone.0034498. 22496820 PMC3320637

[53] Croucher NJ, Page AJ, Connor TR, Delaney AJ, Keane JA, Bentley SD, Parkhill J, Harris SR 2015 Rapid phylogenetic analysis of large samples of recombinant bacterial whole genome sequences using Gubbins. Nucleic Acids Res 43:e15. doi:10.1093/nar/gku1196. 25414349 PMC4330336

[54] Tamura K, Stecher G, Peterson D, Filipski A, Kumar S 2013 MEGA6: molecular evolutionary genetics analysis version 6.0. Mol Biol Evol 30:2725–2729. doi:10.1093/molbev/mst197. 24132122 PMC3840312

[55] Deshpande NP, Wilkins MR, Mitchell HM, Kaakoush NO 2015 Novel genetic markers define a subgroup of pathogenic *Escherichia coli* strains belonging to the B2 phylogenetic group. FEMS Microbiol Lett 362:pii=fnv193. doi:10.1093/femsle/fnv193. 26459886

