# Supplementary material for: Fine-Scale Structure Analysis Shows Epidemic Patterns of Clonal Complex 95, a Cosmopolitan Escherichia coli Lineage Responsible for Extraintestinal Infection
Source: mSphere. 2017 May 31;2(3):e00168-17. doi: 10.1128/mSphere.00168-17 (PMC5451516; doi:10.1128/mSphere.00168-17)
Supplement: TABLE S2 [file sph003172295st2.docx]

**Supplemental Table 2**. Nucleotide sequence of genes found to be unique to one of the four CC95 subgroups.

| CC95 Subgroup | Nucleotide Sequence |
| --- | --- |
| A | >Eh660v1_350050  ATGAAAAGAAGTGAAATCAGGAAAGCACTGGAAGCATGGTTTGATGTTGAACGCTATGAGGCGATAGAGAAACTCACCTTACAGCATTTTTATGTTGAGGTTGAGCGGCGCATCTTGGCTTACAGAATGTTGTTGAGCCGGAACACCATACCTACCTTTAACCGATTAATGCTGGATGATTACCGCAACAAGATCCTCAGCGGTGAAATCTTTTTCAGTGGTGATACCGCTACTCTTGGACATGAGCTTGCCAGAACCTACGCAGTAAATCCTACGACACGAAGCCACGCGCAATTTTATGCGAAAACGCTGGCATTAACCGAAGCCACGCCTGAACTCTCTGGGTTAAGCCAGTCTGAATTTCTGTCCGAGTATCTGAAAGAGACATCCCTTAATAATCTTTCACGTATTACTGTCGATATTCATCTGGAAGAAGCATCAACCGAAGAGATCATTGAACATCTGAAAGTGTTGATCCCCCGATGGAAGAGACAGCTTAAGATGAAGTCTCCAGCGCCAAGAGAGTATCGTTTTGGGAAAAGCACATTCAGAAAAATTATTGAATATCGTCTTATCCCGATGATGGACCTGATCTTCTGGGGAGAAGATAACGGCATTAAAATCCCGCTTTCACTGATTTCCTCGCTGCTTCACGAGGACAGCGACAACGATCGTGATGAAGGAATGCTAAAGGCAACGGACTACCCGTTAGCAATGGCTTTTCTGACGGATGCGAGCTATCTGAAATCGCTTGAAGATTACATGATGGAAAACAATCACTTGAAAGACTCCCCTGTTGAAAAGCATGTAGAAGACGACAGGAATAAGAAAAAATAA |
| B | >Er527v1_680030  ATGATTTCAATACTTACACCTACTTTTAATCGGCAACATACTTTATCAAGGCTATTCAATTCTCTTATATTACAAACTGATAAAGATTTTGAGTGGATAATAATTGATGATGGTAGTATAGATGCAACAGCGGTACTTGTAGAAGATTTTAGAAAAAAATGTGATTTTGACTTGATTTATTGCTATCAGGAAAATAATGGTAAGCCCATGGCTTTAAACGCTGGTGTTAAAGCTTGTAGAGGCGATTATATCTTTATTGTTGACAGTGATGATGCACTAACTCCCGATGCCATAAAATTAATTAAAGAATCAATACATGATTGCTTATCTGAGAAGGAAAGTTTCAGCGGAGTCGGTTTTAGAAAAGCATATATAAAAGGGGGGATTATTGGTAATGATTTAAATAATTCTTCAGAACATATATACTATTTAAATGCGACTGAGATTAGCAATTTAATAAATGGTGATGTTGCATATTGTTTTAAAAAAGAAAGTTTGGTAAAAAATCCATTCCCCCGTATAGAAGATGAAAAATTTGTTCCAGAATTATATATTTGGAATAAAATAACTGACAAGGCGAAGATTCGATTTAACATAAGCAAAGTTATATATCTTTGTGAGTATCTTGATGATGGTCTTTCTAAAAATTTCCATAACCAGCTTAAAAAATACCCAAAGGGGTTTAAGATTTATTACAAAGATCAAAGAAAACGAGAGAAAACTTATATAAAAAAAACAAAGATGCTAATTAGATATTTGCAATGTTGTTATTATGAGAAAATAAAATGA |
| C | >Eh252v1_100119_C3_conserved_hypothetical_protein  ATGGAACTTAGATTAAAAAATGTTACCAGTTATAAGAAAGACGCCTTCACAATACTGAATCTTGAAAAAAGAGTAAACATACTATACGGCCAAAATGGTTGTGGCAAATCTACCATTTCAAATTATTTCTACGACTCTACACACACAGATTATAACGAGTGTGAATGTACCCTTCTTGACAATTACAAACCTCTTGTTTACAACACTAAATTTATTGAAGATAACTTCTATAATGCACAGGAACAAAAAGGTGTTTTTACATTAAGTAAAGAAAATGCAGATATTGAAAAACAATTAATTGAAAAAGAAGCAATTAAACAGCAACTCACAGAGCAATATAGACAAAAAAAAGGAGTGTTATTGAAATTAGAAGCAGAAATGGAAAAAAAAGAAAATGAATACATCGAATCGATCTGGAAAAAAACAGAACCAATCAGAAACTCTGTACTAAAAACCTTAATGAAAGGTAAAGTTGGCTATAAAAGACCTTTTTTTGAGATGATAAAATCATCCTCACCAATCACCTTTATTAATTTAAATGATATAGCAAAAGAATACTCTATACTATTAGAAAATAGAAACAATGAAACCGCATTAATAACTCCTTTACATCCATATACCCCATCAATTGATGATGTAAATACATTAGCAACACCTATAATAGATACAAGCAATAGCTATTTATCAGAAACCATTAATAAACTTCATAATCTTGACTGGGTAAAAAAAGGTAAAGAACATTATTTAGACGGTTCTACATGTCCGTTTTGTCAGGAAAAAACTATTAGTCACGAATTTTTAAATGCCCTTAAATCAATATTTGACGAAAGTTATTCAAAGAAAGTAAAACAGCTCTCAGATATAAAGTCAGTTTATGAGAAAAATACCATATATCATTTTAACGAAATACAAAACAATATATCATCATGTGCACTAATCAATGAGAAAGATAAAGAGAACATTATTTCCAATATTAAAATATTGCAAAACATTGCAGAAAAAAATTTAATAAACATTATTGATAAGATCAACAATCCATCAACATGCGTTACATTAGAAATTGATAAAGAACTAGAAAATCATGTTCAGGAAAGCATAGGCGCTGTTAACAAGAAAATAAGAGACATAAATGATAAGGTTAAAAAACTCAAAGAGTATGAGAATAAAATCGGAAAACAAGTTTGGGGGGCTCTCCATGCTTTTTGCAGCGATGATTTAGAGAATTTATCCAAACACAAAGAAAAATTCTTTGATATCAAAAAGAAAGTTCATGATGAATTAAATAATATTGAGCAACAAGGAAAAGAAAATAATCTCACAATAAAAGCATTGCGAGATAAAATATCTAACATTGATTCTACGATAGACTCAATAAACACCCATTTAAAAAATCTAGGGATCTCTGGTTTTTGTATTGAAAAACATGCAGAAAGAAAAGATATGTACATTATATCTCGACCAGGGCATACAAAAAACCAAAATGTTTATAGATCTCTTAGCGAAGGTGAAAAAACACTAATAACGTTTCTTTATTTTTTAGAGTGCTGTAAAGGTAAAACCGATAAAAATGATACCGATAGCAGGGATATTTTTATTGTTATTGACGATCCAATATCTAGTCTATCGCAAAACTATGTCTACGACATCGCATCAATTATTCATCACCACATTATAAAAAATGAATTAACAAAGAAGGTATTAATCCTTACTCACAATCTTTATTTCTTTCATGAACTTATTAAGCTATCTCCTAAAAGCAGAGAAGACAAACTATTTAAGAAAAACTATTATCTAGGTAGAGTAACGAAAAATGATTTTAGTATAATTACTGAAATACAAAAAAACAGCATACAGAATGAATATCAATCTTTGTGGCAAATATTAAAAGATGCAAAAGATGGCAAAGCAAACAAAATCATAATACCGAATATTATGAGAAACATACTAGAATATTATTTTGCATTTGTTCATAGAACAGATGCTCTACAAGATGAATTAACAAAGTTGGCACGCGACGATAGCAACAGTGATTTCAAAGCATTCTACAGATACATTAACAGAGGTTCTCACTCTGATGCGGTAAATATTACTGATATGGGCGACATAGACCCAGATAAATACCTAAAACAATTGAGAACAATATTTTCAGCTACTGGTGACGAAATGCATTATTTAAAAATGATGGACGAACTAGAAGAGGAAAACGTTACCGCTTAG |
| D | >ECS88_4838 ATGGTCACTAAAAAACAATCTCGCGTTCCAGGTCGTCCCAGACGTTTCGCTCCTGAGCAGGCAGTCTCTGCGGCAAAAGTGCTTTTTCACCAAAAAGGTTTCGATGCTGTCAGTGTTGCTGAAGTTACTGATTATCTTGGTATTAACCCCCCGAGCCTCTACGCGGCTTTTGGCAGTAAGGCTGGGTTATTTAGCCGTGTACTCAATGAATATGTCGGTACGGAAGCTATTCCGCTTGCCGATATTCTTCGTGATGATCGTCCGGTAGGCGAGTGCCTGGTTGAGGTATTAAAAGAAGCGGCGCGCAGATATAGCCAAAACGGCGGCTGCGCTGGCTGTATGGTTCTTGAAGGTATTCATAGTCATGATCCACAAGCGCGTGATATTGCCGTTCAATATTATCACGCCGCAGAAACGACCATTTATGACTATATCGCCAGGCGGCATCCACAAAGCGCACAATGTGTGACTGATTTTATGAGTACCGTGATGTCAGGGCTTTCTGCGAAGGCACGGGAGGGGCACTCCATCGAACAACTCTGTGCAACAGCTGCACTGGCGGGGGAAGCGATAAAAACTATTCTCAAGGAGTGA |
| E | >gi_535555168\|  ATGTCTATCAGTTCAGTAATCAAATCATTACAAGATATTATGCGCAAAGATGCCGGTGTGGACGGCGATGCGCAGCGTCTCGGTCAGCTCTCCTGGCTACTGTTTTTGAAAATCTTCGATGCCCAGGAAGAGGCGCTGGAACTGGAGCAGGATAACTATCAATATCCGATCCCACAGCGTTATTTATGGCGCAGTTGGGCCGCAAACGCTCAGGGCATTACCGGTGATTCCCTGCTGGAATTCGTTAATGATGATCTGTTCCCGGCGTTAAAAAACCTCACTGCGCCTATCGATAAAAACCCACGCGGCTACGTGGTAAAGCAGGCGTTCAGCGATGCCTATAACTACATGAAAAACGGTACGCTACTGCGTCAGGTGATCAACAAGCTGAACGAAATTGACTTTACCAGCGCCAGCGAACGGCATCTGTTTGGTGATATTTACGAACAGATCCTTAAAGATCTGCAATCTGCGGGCAATGCGGGCGAATTCTATACTCCACGCGCCGTCACTCGCTTTATGGTGGATCGCGTTGATCCGAAACTCGGCGAATCCATTATGGACCCGGCCTGCGGTACGGGCGGTTTTCTTGCCTGCGCATTTGATCATGTAAAGAACAAATACGTGAAGAGCGTCGCCGATCATCAGACGCTGCAACAACAGATCCACGGTGTTGAGAAAAAACAGCTTCCGCACCTGCTGGCGACCACCAATATGCTGCTACACGGCATTGAAGTGCCAGTGCAAATTCGTCACGACAACACTCTGAACAAACCGCTTTCCTCCTGGGATGAGCAACTGGATGTCATTGTTACCAACCCGCCGTTTGGTGGCACGGAAGAAGACGGTATTGAGAAGAACTTTCCAGCAGAGATGCAAACCCGCGAAACGGCGGATTTGTTCCTGCAACTGATTGTGGAAGTACTGGCGAAAAACGGTCGTGCGGCGGTGGTATTGCCGGATGGCACGCTATTTGGCGAAGGCGTTAAAACCAAAATCAAAAAGCTGCTTACCGAAGAGTGCAATCTGCATACCATCGTGCGTTTACCGAATGGTGTGTTTAACCCCTATACCGGCATTAAAACCAACCTGTTGTTCTTTACCAAAGGTCAGCCAACCAAAGAGATTTGGTTCTATGAGCATCCGTATCCGGCGGGCGTGAAAAATTACAGTAAAACCAAGCCGATGAAGTTTGAAGAGTTTCAGGCGGAGATCGACTGGTGGGGTAACGAGGCCGATGGTTTTGCCAGCCGCGTCGAGAATGAGCAGGCGTGGAAAGTCAGCATTGATGATGTCATTGCGCGCAACTTCAATCTGGATATTAAAAACCCACATCAGGCGGAAACCGTTAGCCATGATCCGGACGAACTGTTAGCGCAGTATGCAAAACAGCAGGCGGAGATCCAGACGCTGCGTAATCAACTGCGCGATATTCTTGGCGCTGCGCTGTCTGGCAAGGAGGTTAACTAA |
