# Supplementary material for: Fine-Scale Structure Analysis Shows Epidemic Patterns of Clonal Complex 95, a Cosmopolitan Escherichia coli Lineage Responsible for Extraintestinal Infection
Source: mSphere. 2017 May 31;2(3):e00168-17. doi: 10.1128/mSphere.00168-17 (PMC5451516; doi:10.1128/mSphere.00168-17)
Supplement: TABLE S3 [file sph003172295st3.docx]

**Supplemental Table 3.** Primer sequences and characteristics used for the classification of CC95 strains in 4 subgroups.

| Subgroup  Detected | Primer Name | Primer sequence | Tm °C | GC% | Product Size, bp |
| --- | --- | --- | --- | --- | --- |
| A | gpA F  gpA R | 5’-CAACAAGATCCTCAGCGGTG-3’  5’-GCGATTTCAGATAGCTCGCA-3’ | 59.4  57.3 | 55.0  50.0 | 565 |
| B | gpB F  gpB R | 5’-CACTAACTCCCGATGCCATA-3’  5’-ATCGAATCTTCGCCTTGTCA-3’ | 57.3  55.3 | 50.0  45.0 | 315 |
| C | gpC F  gpC R | 5’-AGACGCCTTCACAATACTGA-3’  5’-GAAGGGTACATTCACACTCG-3’ | 55.3  57.3 | 45.0  50.0 | 137 |
| D | gpD F  gpD R | 5’-AGTGCCTGGTTGAGGTATTA-3’  5’-CCTGACATCACGGTACTCAT-3’ | 55.3  57.3 | 45.0  50.0 | 226 |
| E | gpE F  gpE R | 5’-TGGAACTGGAGCAGGATAAC-3’  5’-CTTCCACAATCAGTTGCAGG-3’ | 57.3  57.3 | 50.0  50.0 | 786 |
